# Panton-Valentine Leukocidin associated with *S*. *aureus* osteomyelitis activates platelets via neutrophil secretion products

**DOI:** 10.1038/s41598-018-20582-z

**Published:** 2018-02-01

**Authors:** Silke Niemann, Anne Bertling, Martin F. Brodde, Anke C. Fender, Hélène Van de Vyver, Muzaffar Hussain, Dirk Holzinger, Dirk Reinhardt, Georg Peters, Christine Heilmann, Bettina Löffler, Beate E. Kehrel

**Affiliations:** 1 Institute of Medical Microbiology, University of Muenster, Muenster, Germany; 2 Department of Anaesthesiology, Intensive Care and Pain Medicine, Experimental and Clinical Haemostasis, University of Muenster, Muenster, Germany; 3 Interdisciplinary Center for Clinical Research (IZKF) Muenster, Muenster, Germany; 4 Department of Pediatric Hematology-Oncology, University of Duisburg-Essen, Essen, Germany; 5 Cluster of Excellence EXC 1003, Cells in Motion, Muenster, Germany; 6 Institute of Medical Microbiology, Jena University Hospital, Jena, Germany

## Abstract

Globalization and migration promote the spread of Panton-Valentine leukocidin (PVL)-positive *Staphylococcus aureus* strains. The toxin PVL is linked to the development of thrombosis in association with osteomyelitis. The mechanisms by which PVL drives thrombosis development are however still unknown. We demonstrate that PVL-damaged neutrophils activate platelets via neutrophil secretion products, such as α-defensins and the myeloperoxidase product HOCl, as well as the formation of HOCl-modified proteins. Neutrophil damage by PVL is blocked by anti-PVL-antibodies, explaining why especially young osteomyelitis patients with a low antibody titre against PVL suffer from thrombotic complications. Platelet activation in the presence of PVL-damaged neutrophils is prevented by α-defensin inhibitors and by glutathione and resveratrol, which are both inhibitors of HOCl-modified protein-induced platelet activation. Remarkably, intravenously infused glutathione also prevents activation of human platelets in an *ex vivo* assay. We here describe a new mechanism of PVL-neutrophil-platelet interactions, which might be extrapolated to other toxins that act on neutrophils. Our observations may make us think about new approaches to treat and/or prevent thrombotic complications in the course of infections with PVL-producing *S*. *aureus* strains.

## Introduction

Although deep vein thrombosis (DVT) occurs very rarely in children^1^ more and more cases have been reported in recent years in connection with osteomyelitis, with DVT occurring in 10% of community-acquired acute haematogenous osteomyelitis cases^2^. Interestingly, this complication is more frequent in young patients than in adults. *Staphylococcus aureus* is the predominant causative agent for osteomyelitis in children^3^ and, although the mechanisms are unknown, there is increasing evidence for an association of Panton-Valentine leukocidin (PVL)-expressing *S*. *aureus* strains with acute haematogenous osteomyelitis severity^4,5^.

Generally, PVL is linked to community-associated methicillin-resistant *S*. *aureus* (CA-MRSA) infections, particularly of skin and soft tissue^6^, and to highly lethal necrotizing pneumonia, especially in young immunocompetent patients^7,8^. However, methicillin-sensitive *S*. *aureus* strains can carry the PVL genes as well^6^.

In Germany, the prevalence of PVL is still very low^9^, but in other parts of the world, such as Africa, a large proportion of *S*. *aureus* isolates harbour PVL^10^. In the USA, over one third of *S*. *aureus* infection isolates are PVL-positive, with the USA300 clone accounting for 86% of all PVL-positive isolates detected^11^. In times of increasing globalization, travelling and migration lead to a faster spread of - and hence higher infection rates with - PVL-positive strains^12,13^.

PVL is a two-component (LukS-PV and LukF-PV), β-barrel pore-forming toxin^14^. Pore formation occurs in a stepwise fashion. The LukS-PV binds to the complement receptor C5aR, hetero-oligomerization of the S component with the F component then results in the insertion of the hydrophobic stem into the membrane of the target cell that spans the host cell lipid bilayer. The formation of pores leads to cell lysis due to leakage of divalent cations that are essential for cell homeostasis^15^. The main target cells of PVL are polymorphonuclear leukocytes (PMNLs, neutrophils), with high species specificity. PVL targets human as well as – to a lesser extent – rabbit neutrophils, but does not affect neutrophils from mice or Java monkeys^16^. PVL-treated neutrophils show degranulation and oxidative burst reactions and release pro-inflammatory substances such as interleukin (IL)−6, IL-8 and tumour necrosis factor α (TNFα)^17,18^, which are generally thought to contribute to thrombus formation when it occurs in association with PVL-*S*. *aureus* osteomyelitis^19,20^. To further elucidate the underlying pathophysiology, we examined the direct effect of PVL on platelets, and its indirect effects in the presence of neutrophils. We show that platelets are activated secondary to the release of α-defensins and the myeloperoxidase product HOCl from neutrophils, as well as the formation of HOCl-modified proteins. The mechanism identified by this study contributes to our general understanding of the pathophysiology of osteomyelitis, and provides one possible explanation for the development of thrombosis in this setting. Moreover, our findings will hopefully stimulate the re-evaluation of new therapeutic concepts for the treatment and/or prevention of the thrombotic complications in connection with *S*. *aureus* osteomyelitis.

## Results

### PVL only activates platelets in the presence of human neutrophils

**Figure 1:**
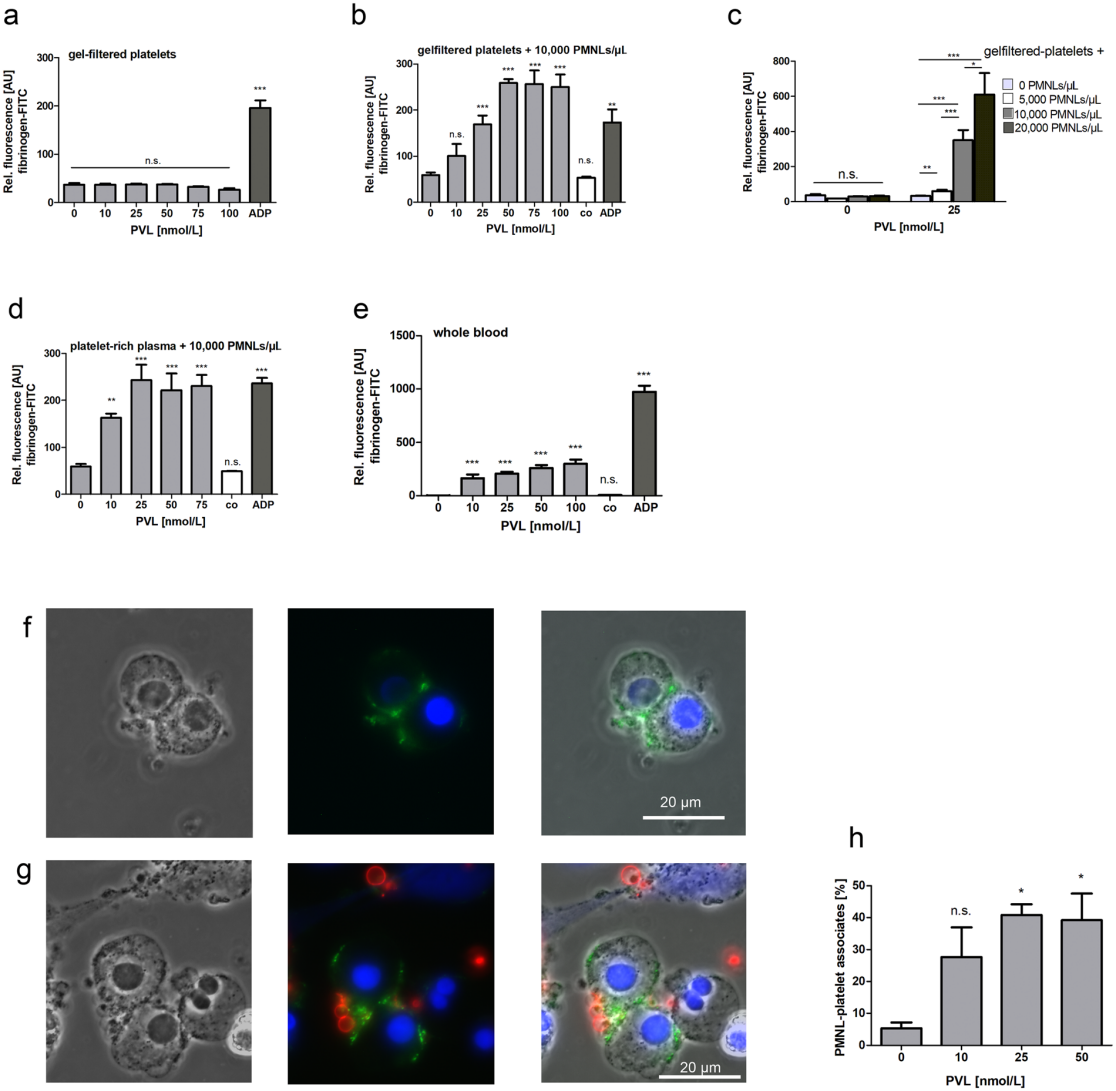
PVL activates platelets only in the presence of PMNLs. Flow cytometry analysis of the effect of PVL on fibrinogen-FITC-binding to gel-filtered platelets (**a**), in the absence or presence of isolated PMNLs (10,000 per µL) (**b**), and in dependence on PMNL-concentration (**c**). Flow cytometry analysis of the effect of PVL on fibrinogen-FITC-binding to platelets in plasma in the presence of PMNLs (10,000 per µL) (**d**). Flow cytometry analysis of the effect of PVL on fibrinogen-FITC binding to anti-CD42a-PE-stained platelets in whole blood (**e**). Activation with 10 µmol/L ADP served as positive and 9.6 µL PBS as vehicle control (co). Fluorescence microscopy of PMNLs (**f**), and PMNLs together with gel-filtered platelets, stained with anti-CD42a-PE (red) (**g**). In each experimental setting for microscopy, cells were incubated with 25 nmol/L FITC-labelled PVL (green) for 1 h. Hoechst33343 stain was used for nucleic acid staining. Phase contrast and merged pictures are shown. For quantification of PMNL-platelet associates, platelets were labelled with CD42aPE. After stimulation of platelets with PVL in presence of neutrophils the percentage of CD42aPE-positive PMNLs was analysed by flow cytometry (**h**). Flow cytometric data represent means + SD of three independent experiments. Statistical significance was analyzed by one-way ANOVA followed by Dunnett’s multiple comparisons test (* p < 0.05; **p < 0.01; ***p < 0.001). Dependence on PMNL concentration was analyzed by one-way ANOVA followed by Bonferroni testing.

Platelet activation is accompanied by conformational changes in the major platelet fibrinogen receptor GPIIb/IIIa, which increases the affinity and binding of GPIIb/IIIa to soluble fibrinogen. We first determined the direct effect of PVL on human platelets by assessing the binding of FITC-coupled fibrinogen to platelets. PVL in concentrations up to 100 nmol/L had no effect on fibrinogen-FITC binding to gel-filtered platelets even after 1 h of incubation (Fig. 1a). By contrast, when gel-filtered platelets were treated with PVL (10–100 nmol/L) in the presence of isolated neutrophils (10,000 per µL), fibrinogen-FITC binding to platelets was dramatically increased (Fig. 1b). Platelet activation with 25 nmol/L PVL in the presence of neutrophils was comparable with the direct activation of platelets with 10 µmol/L ADP. The vehicle control (9.6 µL PBS) was without effect. This indirect effect of PVL on platelet fibrinogen-FITC binding increased significantly with rising neutrophil concentration (Fig. 1c). PVL-induced platelet fibrinogen-FITC binding was also observed using platelet-rich plasma supplemented with 10,000 neutrophils per µL (Fig. 1d) and importantly, also in whole blood (Fig. 1e). Not only recombinant PVL but also the supernatant of a PVL-producing *S*. *aureus* strain (USA300) induced fibrinogen-FITC binding to platelets in the presence of neutrophils. Although supernatant from the corresponding mutant USA300ΔPVL strain stimulated fibrinogen-FITC binding to platelets as well, this effect was nearly one-third reduced in comparison with the wildtype strain (Supplementary Fig. S1a). Vehicle controls (2% (v/v) brain heart infusion (BHI) ± 150 µg/mL spectinomycin) had no effect.

### PVL directly targets human neutrophils, but not human platelets

**Figure 2:**
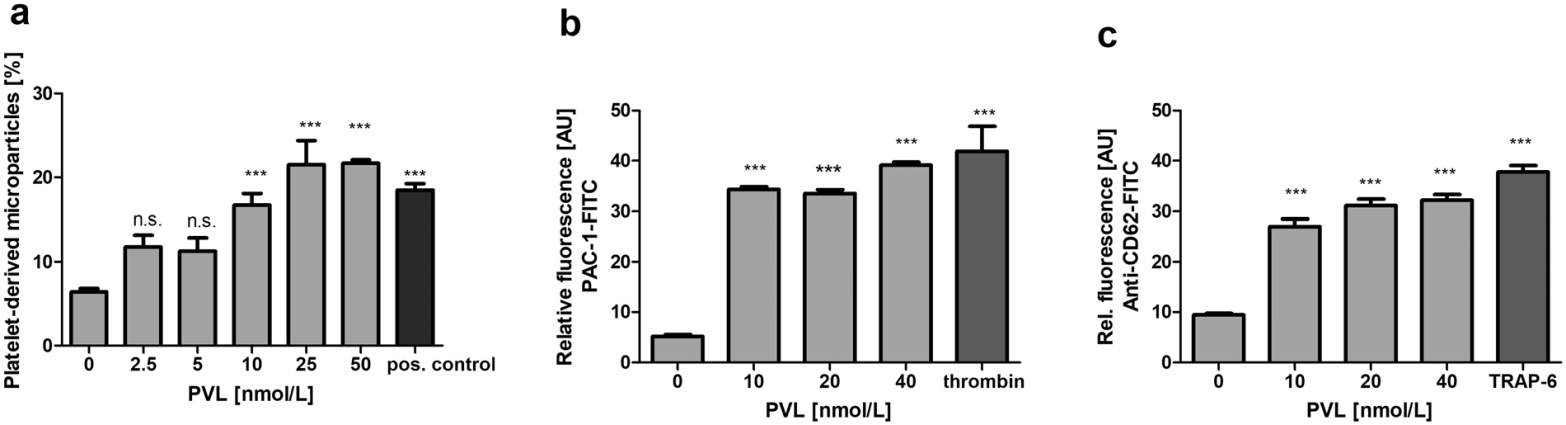
In combination with PMNLs PVL induces formation of platelet-derived microparticles, GPIIbIIIa activation and α-granule release. Flow cytometric analysis of the PVL-induced generation of microparticles by gel-filtered platelets in the presence of isolated PMNLs. Microparticles were identified by reduced size and expressed as percentage of all anti-CD42a-PE-positive events measured. Microparticle generation evoked by 0.5 µg/mL collagen and 2 U/mL thrombin served as positive control (pos. control) (**a**). Flow cytometric analysis of the effect of PVL on gel-filtered platelets in the presence of isolated PMNLs: PAC-1-FITC binding (positive control 0.1 U/mL thrombin) (**b**), and anti-CD62P-FITC binding, (positive control 50 µmol/L TRAP-6) (**c**). Flow cytometric data represent means + SD of three independent experiments. Statistical significance was analyzed by one-way ANOVA followed by Dunnett’s multiple comparisons test (* p < 0.05; **p < 0.01; ***p < 0.001).

Fluorescence microscopy showed that FITC-coupled PVL binds to isolated human neutrophils (Fig. 1f), but not to gel-filtered platelets (Fig. 1g). Simultaneous incubation of neutrophils and gel-filtered platelets with PVL-FITC revealed associate formation of PVL-FITC-covered neutrophils with gel-filtered platelets (Fig. 1g), which could also be demonstrated by flow cytometry (Fig. 1h). In combination with neutrophils PVL induced the formation of platelet-derived microparticles (Fig. 2a) as well as PAC-1 binding to platelets, indicating activated GPIIb/IIIa (Fig. 2b), and CD62P (platelet surface P-selectin) expression on the platelet surface, revealing platelet activation with α-granule secretion (Fig. 2c).

**Figure 3:**
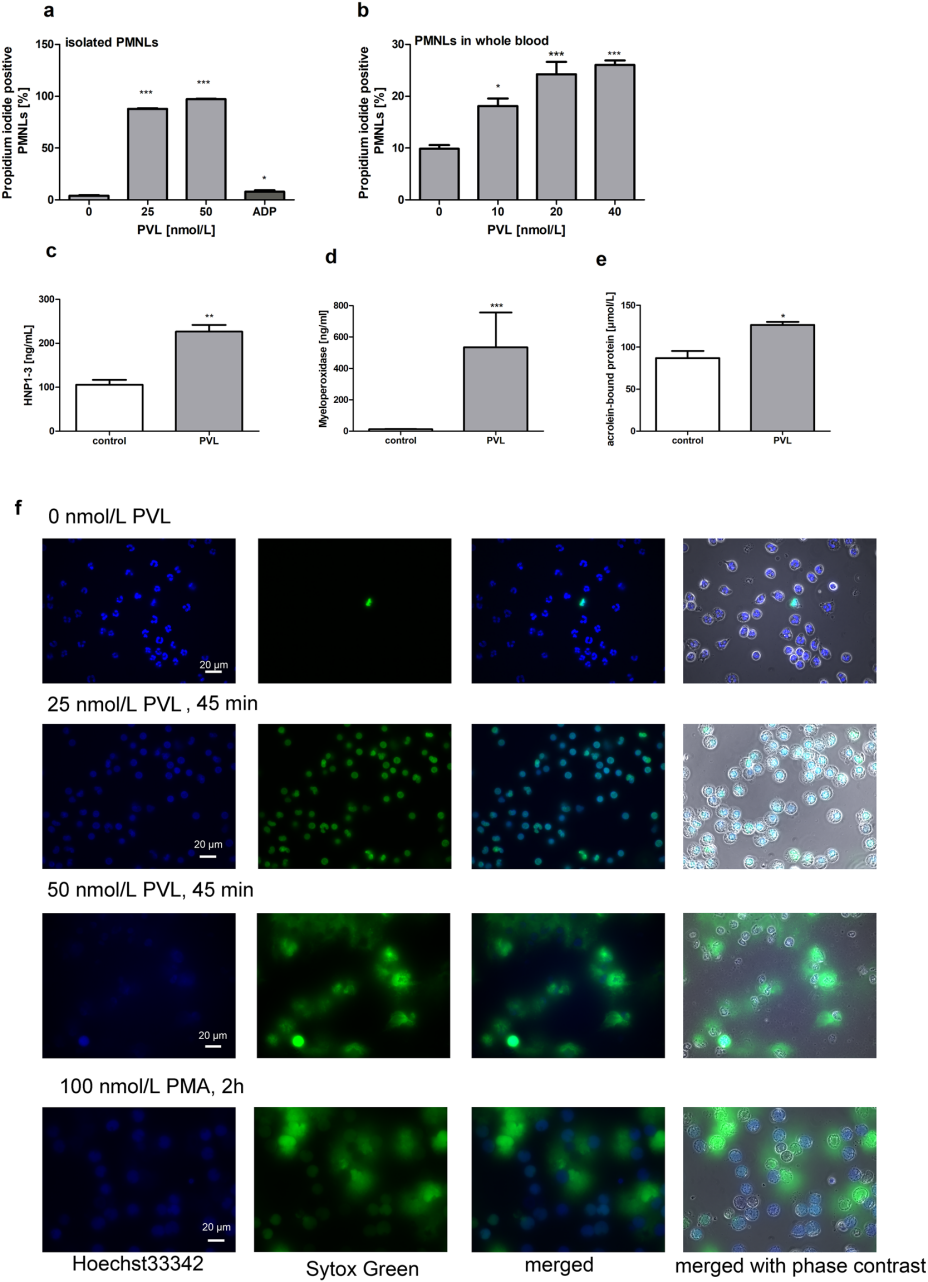
PVL induces damage of PMNLs. PVL-induced lysis of isolated PMNLs (**a**), and of PMNLs in whole blood (gated by size scatter and stained with CD16b) (**b**), was analysed by flow cytometry by propidium-iodide staining. PVL-induced release of human neutrophil peptides HNP1–3 (**c**), myeloperoxidase (**d**), and acrolein-bound proteins (**e**) by isolated PMNLs was assessed by ELISA. Flow cytometric data represent means + SD of three independent experiments. Statistical significance was analyzed by one-way ANOVA followed by Dunnett’s multiple comparisons test (* p < 0.05; ** p < 0.01; *** p < 0.001). Freshly isolated human neutrophils were incubated with 25 nmol/L PVL for 45 min, followed by staining with Hoechst33342 (blue) and Sytox Green (green) to analyse cell-membrane damage and NET formation. PMA served as positive control for NET formation. An overlay of the signals from Hoechst33342 and Sytox Green staining is shown (blue and green, merge) as well as an overlay of the signals with the image from phase contrast microscopy (**f**).

Propidium iodide staining revealed that isolated human neutrophils and neutrophils in whole blood are damaged by PVL, but not by the platelet activator ADP (Fig. 3a,b). Treatment of neutrophils with supernatant of the PVL producing *S*. *aureus* strain USA300 had a comparable damaging effect to direct PVL treatment. This detrimental effect could be reduced by approximately 25% with USA300ΔPVL supernatant (Supplementary Fig. S1b). No neutrophil lysis was seen with the vehicle controls. PVL additionally induced the secretion of α-defensins HNP1–3 and myeloperoxidase into the supernatant of isolated human neutrophils (Fig. 3c,d). The amount of acrolein-bound proteins in neutrophil-conditioned medium, a measure of oxidative damage associated with myeloperoxidase catalysis^21^ or lipid peroxidation^22^, was also increased by PVL (Fig. 3e). Defensin-release by PVL-treated neutrophils, as well as oxidative reactions detected by an antibody directed against the acrolein derived adduct *Nε*-(3-formyl-3,4-dehydropiperidino)-lysine (FDP-lysine) (see below), were also visualised by fluorescence microscopy (Supplementary Fig. 2). In addition, PVL induced the release of neutrophil extracellular traps (NETs) which was comparable to PMA stimulation at 50 nmol/L PVL (Fig. 3f).

### Neutrophil damage by PVL and subsequent platelet activation is inhibited by antibodies against PVL

**Figure 4:**
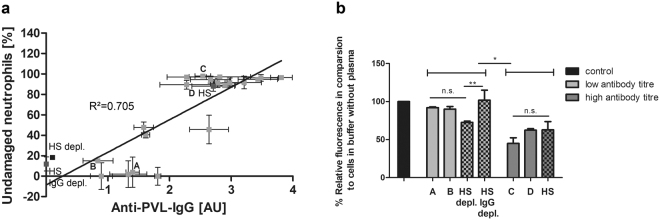
Inhibiting effect of human plasma on neutrophil lysis and subsequent platelet activation by PVL correlates with the amount of antibodies against PVL in the plasma/serum. Human neutrophils were incubated with 2.5% human plasma obtained from 18 different blood donors, or with 2.5% human serum (HS), human serum IgG depleted (HS IgG depl.) or human serum anti-PVL antibody depleted (HS depl.). Cell damage after incubation with PVL (5 nmol/L) was measured by flow cytometry assessment of propidium-iodide staining. Antibodies against PVL in the plasma/ serum were measured by ELISA (**a**). Data show mean ± SD from 3 different experiments. Pearson’s correlation coefficient was used to determine the relationship of percentage of undamaged neutrophils and anti-PVL-antibody titre. Inhibiting effect of plasma/serum from donors with a high titre of anti-PVL antibodies on platelet fibrinogen-binding in the presence of isolated human neutrophils: PVL (25 nmol/L)-induced binding of fibrinogen-FITC to gel-filtered platelets in the presence of isolated human PMNLs (10,000 per µL) and 10% plasma/serum from donors with high (donors C and D) or low anti-PVL titers (donors A and B) was measured by flow cytometry and compared to the control without plasma (100%). (**b**) Data show mean ± SD from 3 different experiments, one-way ANOVA followed by Bonferroni testing (*p < 0.05; **p < 0.01).

Patients infected with PVL-positive *S*. *aureus* strains have been shown to produce specific anti-PVL antibodies^23^. Recently, we demonstrated that human serum protects neutrophils against PVL-induced lysis, in direct correlation with the level of anti-PVL antibodies in the serum^24^. We now tested the effect of human plasma, as well as of human serum and IgG-depleted human serum, on PVL-induced lysis of human neutrophils and subsequent platelet activation. Human neutrophils were incubated with 2.5% human plasma obtained from 18 different blood donors. PVL (5 nmol/L)-induced cell damage was determined by propidium iodide staining and FACS analysis. Simultaneously, antibodies against PVL in the plasma were measured by specific ELISA. Plotting the percentage of undamaged neutrophils against the antibody titre, the coefficient of determination was R^2^ = 0.7046 (Pearson’s r = 0.832, p = 0.01), consistent with a linear relation between the amount of antibodies against PVL, and the inhibiting effect of the plasma (Fig. 4a). Furthermore, addition of 2.5% human plasma from two donors with a high anti-PVL titre significantly inhibited PVL-induced binding of fibrinogen-FITC to gel-filtered platelets in the presence of isolated human neutrophils, whereas plasma from donors with low anti-PVL titre had no effect (Fig. 4b). Similarly, human serum with a high anti-PVL titre inhibited platelet activation by PVL in the presence of neutrophils, while commercially available IgG-depleted human serum had no effect. A similar result was obtained using human serum that had been specifically depleted of anti-PVL antibodies.

### Platelet activation by PVL-treated neutrophils is blocked by α-defensin inhibitors

**Figure 5:**
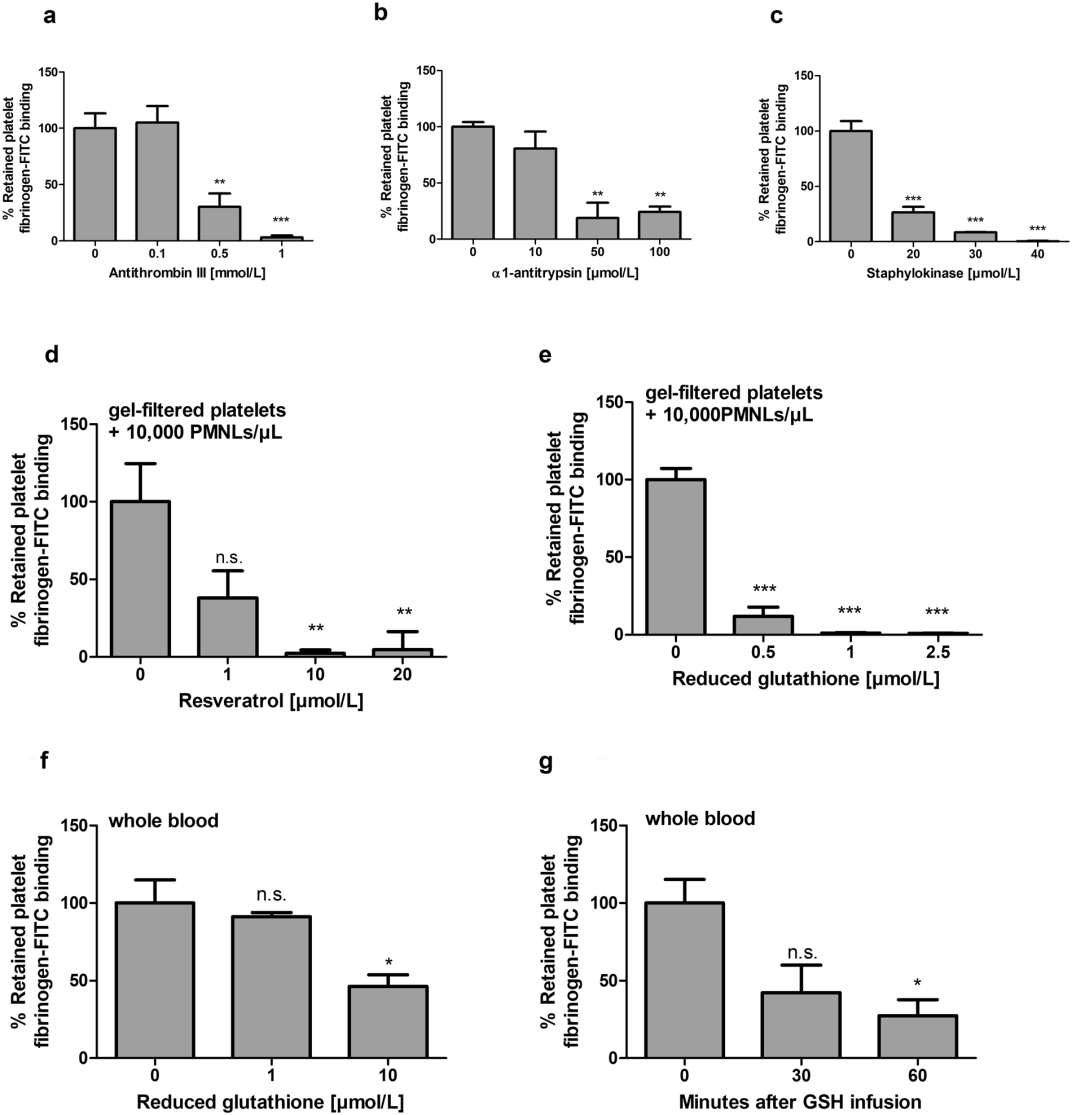
PVL-induced platelet activation is inhibited by common defensin inhibitors such as serpins, and staphylokinase (SAK) and by glutathione and resveratrol. Flow cytometric analysis of the effect of antithrombin III (**a**), α1-antitrypsin (**b**), and staphylokinase (**c**) on PVL-induced platelet fibrinogen binding. Gel-filtered platelets were incubated with PVL (25 nmol/L) in the presence of 2 mmol/L CaCl_2_ and 10,000 isolated PMNLs per µL for 60 min at room temperature. Flow cytometric analysis of the effect of resveratrol on PVL (25nmol/L)-induced platelet fibrinogen-FITC binding to gel-filtered platelets in the presence of 10,000 isolated PMNLs per µL (**d**). Flow cytometric analysis of the effect of reduced glutathione on PVL (25 nmol/L)-induced platelet fibrinogen-FITC binding to gel-filtered platelets in the presence of 10,000 isolated PMNLs per µL (**e**) and anti-CD42a-PE-stained platelets in whole blood (**f**). The *ex vivo* effect of reduced glutathione on PVL (25 nmol/L)-induced platelet fibrinogen-FITC binding was analysed by flow cytometry using propidium iodide staining (**g**). Whole blood from three different blood donors, taken before and 30 min or 60 min after intravenous infusion of reduced glutathione, was incubated *ex vivo* with PVL for 60 min at room temperature. Flow cytometric data represent means + SD of three independent experiments. Statistical significance was analyzed by one-way ANOVA followed by Dunnett’s multiple comparisons test (*p < 0.05; **p < 0.01; ***p < 0.001).

We next set out to identify which of the substances released by PVL-lysed neutrophils drive platelet activation. Primary candidates were the α-defensins HNP1–3, which are increased in the supernatant of PVL-treated human neutrophils, and have been shown by us and others to activate human platelets in clinically relevant concentrations^25,26^. This activation is inhibited by serine protease inhibitors (serpins) in a thrombin-independent manner^25^. Therefore, we tested the effect of physiologically relevant concentrations^27,28^ of the serpins antithrombin III and α_1_-antitrypsin on PVL-induced platelet activation in the presence of isolated neutrophils. Fibrinogen-FITC binding to gel-filtered platelets induced by 25 nmol/L PVL in the presence of neutrophils was blocked by 0.5 and 1 mmol/L antithrombin III (Fig. 5a) and 10–100 µmol/L α_1_-antitrypsin (Fig. 5b), but α_1_-antitrypsin did not block ADP activation of platelets (Supplementary Fig. S3). HNPs have been reported to form complexes with staphylokinase (SAK), a thrombolytic protein secreted by *S*. *aureus*, which acts by enhancing the affinity of tissue-type plasminogen activator (tPA) for plasminogen and facilitating its proteolytic activation. The complexes are characterized by a six-fold excess of defensin and by loss of function on both sides^29,30^. Accordingly, excess SAK blocked the effect of HNPs on platelet activation (Supplementary Fig. S4). Addition of SAK in concentrations that attenuated HNP-induced platelet activation also inhibited PVL-induced fibrinogen binding to gel-filtered platelets in the presence of isolated neutrophils (Fig. 5c). Thus HNPs, as one group of substances released by neutrophils upon PVL damage, are important triggers for subsequent platelet activation.

### Platelet activation by PVL-treated neutrophils is blocked by glutathione and resveratrol

In addition to HNPs, PVL-stimulated neutrophils also secreted substantial amounts of myeloperoxidase. The myeloperoxidase product HOCl reacts with protein moieties and lipids, generating HOCl-modified-albumin, LDL and other species that can potently activate platelets^31,32^. HOCl also leads to the production of the α, β- unsaturated aldehyde acrolein^21^. One major target of acrolein is the nucleophilic amino acid lysine in proteins, forming Nε-(3-formyl-3,4-dehydropiperidino) lysine (FDP-lysine)^22^. This reacts with free thiol groups in, among other proteins, glutathione (GSH), a known scavenger of myeloperoxidase reaction products^33^. We have observed that glutathione and a number of polyphenols such as resveratrol can counteract the platelet-activating effects of HOCl-modified proteins (unpublished data, presented in abstract form)^34^. In addition, resveratrol, a natural antioxidant in grapes, has been shown to inhibit neutrophil myeloperoxidase by direct interaction with the enzyme^35^. In this study, resveratrol (1–10 µmol/L) blocked PVL-induced fibrinogen-FITC binding to gel-filtered platelets in the presence of neutrophils (Fig. 5d), as did physiologically relevant concentrations^36^ of reduced glutathione (GSH, Fig. 5e). GSH similarly suppressed PVL-induced fibrinogen-FITC binding to anti-CD42a-PE-stained platelets in whole blood (Fig. 5f), and was moreover effective in whole blood obtained from blood donors 30 and 60 min after intravenous administration of GSH (Fig. 5g). Neither resveratrol nor GSH blocked ADP activation of platelets (Supplementary Fig. S3).

We were able to obtain blood from one young patient diagnosed for chronic granulomatous disease (CGD), who fails to produce a significant oxidative burst upon neutrophil stimulation. In this patient, the proportion of cells having produced reactive oxygen radicals upon stimulation with N-formyl-Met-Leu-Phe (fMLP) was 2% (reference range 30–78%). Platelet activation (in PRP from the patient or a healthy control) in the presence of PVL-stimulated patient neutrophils was only modestly reduced in comparison to the effect of PVL-stimulated neutrophils obtained from the healthy donor (Supplementary Fig. S5a,b). Platelet activation by ADP was reduced as well (Supplementary Fig. S5c). ELISA analyses of plasma samples obtained from whole blood after stimulation with either PVL (50 nmol/L) or the positive control PMA (100 nmol/L or 1.6 µmol/L), revealed that neutrophils from the CGD patient were able to secrete α-defensins HNP1–3 and myeloperoxidase, and to produce acrolein and NETs. The samples (neutrophil supernatants and plasma) from patient and control (Supplementary Table 1) showed no marked differences although values obtained in patient samples were consistently slightly lower values than control samples, in keeping with the minimal divergence between patient and control platelet activation by neutrophils from either patient or control.

### Inhibition of PVL-triggered PMNL stimulation by the antioxidants resveratrol and taurine

**Figure 6:**
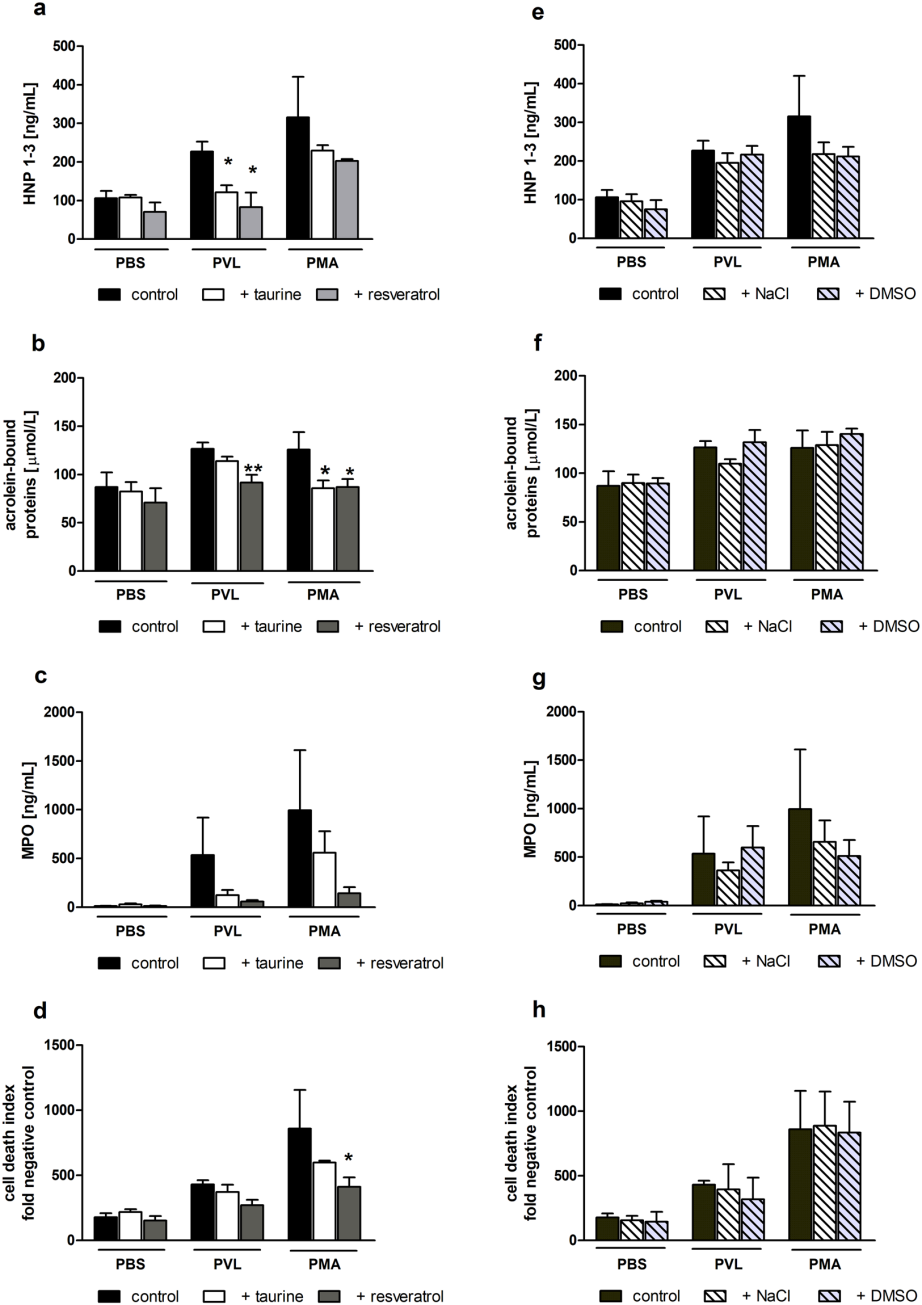
Antioxidants reduce PVL-stimulated release of neutrophil secretion products and NETosis. Supernatants from human neutrophils stimulated ±PVL (25 nmol/L) or PMA (100 nM), in the absence and presence of either taurine (10 mmol/L) or resveratrol (50 µmol/L), for 1 h (**a-d**), or with vehicle controls NaCl (0.9%) and DMSO (0.05% in PBS) for taurine and resveratrol, respectively (**e-h**), were analysed by ELISA for: alpha-defensins HNP1–3 release (**a**, **e**), acrolein-bound protein formation (**b**, **f**), myeloperoxidase (MPO) release (**c**, **g**) or nucleosomes as a surrogate marker for NET formation (**d**, **h**). Data show mean ± SD from 3 independent experiments. Flow cytometric data represent means + SD of three independent experiments. Statistical significance was analyzed by one-way ANOVA followed by Dunnett’s multiple comparisons test (*p < 0.05, vs PVL or PMA without antioxidant).

We next examined the potential inhibitory impact of antioxidants on the direct stimulatory effect of PVL on neutrophils. Conditioned supernatants from isolated neutrophils stimulated with PVL (25 nmol/L, 1 h) or the positive control PMA (100 nmol/L, 1 h) ± each resveratrol or taurine, were analysed by ELISA for HNP1–3, MPO, nucleosome release as markers of NET-formation, and the formation of acrolein-bound proteins. Both antioxidants attenuated the stimulatory effects of both PVL and PMA on neutrophil products (Fig. 6), as well as on platelet activation in PRP exposed to 20% (v/v) of conditioned neutrophils supernatants (Supplementary Fig. S6).

### Platelets are activated by supernatant of phenol soluble modulin α3-treated neutrophils

**Figure 7:**
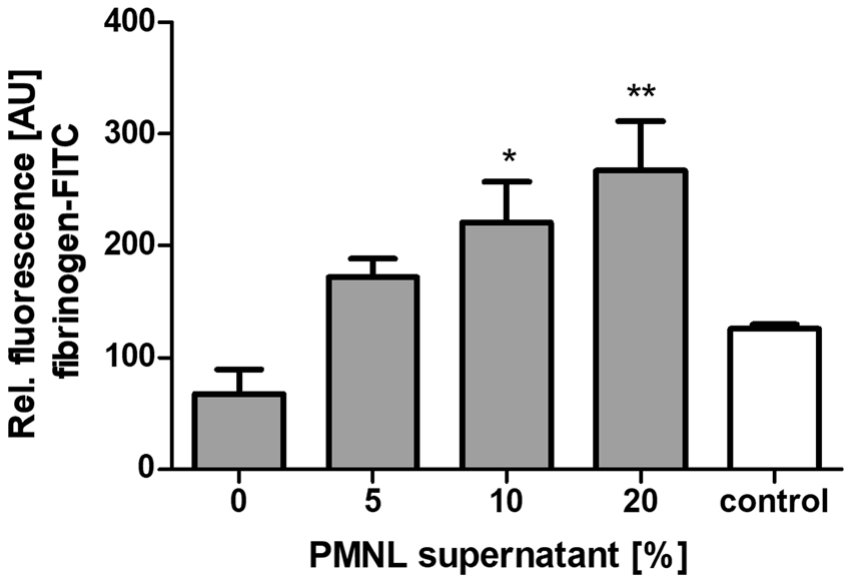
Supernatant from PSMα3 treated neutrophils activates platelets. 10,000 isolated PMNLs/µL were treated with 60 µg/mL PSMα3 for 1 h then centrifuged. Platelets were subsequently incubated with the neutrophil supernatant and fibrinogen-FITC binding was analysed by flow cytometric analysis. Statistical significance was analyzed by one-way ANOVA followed by Dunnett’s multiple comparisons test (*p < 0.05; **p < 0.01).

To extrapolate the indirect platelet-activating effects of PVL to other neutrophil targeting *S*. *aureus* toxins, we examined phenol-soluble modulin α3 (PSMα3) as a representative example. A pilot study with PSMα3 and platelets alone showed that the toxin alone already activates platelets to some extent (Supplementary Fig. S7a). The experimental approach was therefore adjusted. Instead of incubating platelets and neutrophils together with PSMα3, neutrophils were first pre-treated with up to 60 µg/mL of PSMα3, a concentration we previously found to elicit a highly damaging impact on neutrophils^16^. After 1 h of pre-incubation, neutrophils were centrifuged and only the supernatant was added to the platelets. This approach limited the final concentration of PSMα3 to which platelets were exposed to maximally 12 µg/mL, which has only a negligible direct impact on platelet activation. By contrast, the supernatant of PSMα3-treated neutrophils strongly activated the platelets (Fig. 7). The supernatant of ultrasound-treated neutrophils also had an activating impact on the platelets (Supplementary Fig. S7b) which was also blocked by resveratrol or GSH (Supplementary Fig. S7c and d). This demonstrates the validity of platelet activation after neutrophil damage.

## Discussion

PVL secreted by *S*. *aureus* has been linked to severe life-threatening infections, such as osteomyelitis, often accompanied by DVT especially in young patients^5,19,20^. In this study, we examined the interaction between neutrophils, platelets and the *S*. *aureus*-secreted toxin PVL, which might play a central role in thrombosis development. We examined the effect of recombinant PVL on human platelets and neutrophils, at pathologically-relevant PVL concentrations of up to 100 nmol/L (4 µg/mL) as they can be detected in bone abscess samples (0.10–256 µg/mL with a medium concentration of 128 µg/mL)^37^. We observed that PVL bound to isolated human neutrophils but not to platelets, and that neutrophils formed heterotypic associates with platelets when treated with PVL. Increased numbers of circulating platelet-neutrophil associates are often observed in septic patients and might account for the development of multiple organ failure^38^. As described previously, PVL induced lysis of human neutrophils and the release of antimicrobial α-defensins, also named HNPs, and myeloperoxidase^16,17,24^. Recently, we and others were able to show that HNPs activate platelets^25,26^, leading to platelet fibrinogen binding and the formation of platelet-derived microparticles^25^. HNPs have important functions in antimicrobial defence. Release of these peptides in response to PVL might therefore represent the protective reaction of the host to bacterial infection. Indeed, the PVL-producing methicillin-resistant *S*. *aureus* strain USA300 is susceptible to HNP-1 (Supplementary Fig. S8). PVL additionally increased the formation of acrolein-bound proteins in neutrophil-conditioned plasma. Acrolein is generated by human neutrophils via the myeloperoxidase/hydrogen peroxide/chloride system^21^ and by other mechanisms, and has been associated with exacerbated inflammatory and immune responses^39,40^ as well as with ischemic stroke^41^.

We documented no direct effect of PVL on gel-filtered human platelets or human platelets in plasma. PVL stimulated platelet activation solely in the presence of human neutrophils. In this setting, PVL also induced the shedding of microparticles, which is generally only observed upon strong stimulation of platelets, such as with combined thrombin and collagen or HNPs^25,42^. Platelet derived particles have been shown to induce thrombin generation and therefore to be important in thrombosis development^43^.

We propose that the action of PVL on platelets in the pathogenesis of thrombosis in association with *S*. *aureus* osteomyelitis is a two-step mechanism. First, high numbers of neutrophils accumulated at the site of osteomyelitis are lysed by PVL, causing them to release substances such as HNPs or myeloperoxidase, which then in a second step strongly activate platelets, both directly and through formation of FDP-lysine presenting proteins. This constellation of effects then drives thrombosis development through subsequent platelet aggregation and thrombin generation, which in turn leads to further platelet activation and fibrin formation. This indirect action of PVL, mediated via destruction of neutrophils, is similar to the pathogenic mechanisms we have reported for the development of necrotizing pneumonia, where uncontrolled neutrophil cell death due to PVL-expressing *S*. *aureus* strains leads to epithelial destruction^24^. The cytotoxic role of PVL during bacterial infections is further strengthened by results obtained with diluted supernatants of the PVL expressing *S*. *aureus* strain USA300, which functionally resembled the purified PVL, and the significantly less pronounced impact of the corresponding ΔPVL strain compared to the wild type strain. Complete abrogation of platelet activation was not to be expected, given that a number of other direct platelet-activating substances, such as α- toxin^44^ or Eap^45^, are also found in the supernatant of the *S*. *aureus* strains used here.

In accordance with an earlier report^46^, we found that PVL is also able to stimulate neutrophils to release NETs, a component of the endogenous host defence mechanism. However, several *S*. *aureus* strains such as the well-characterised PVL-producing strain USA300, are also able to generate *S*. *aureus* nuclease and can thereby resist killing by NETs^47^. Thus, NET formation might have only a low impact on the overall pathogenic processes we describe. At the same time, these extracellular DNA traps themselves promote thrombosis^48^, and impaired NET degradation is associated with acute thrombotic microangiopathies^49^. Therefore, NET formation might to some extent enhance thrombus formation and stability in DVT occurring in connection with osteomyelitis triggered by a PVL-expressing strain. The role of NET formation in this context is the subject of ongoing studies.

To validate the importance of platelet activating substances released upon neutrophil damage or lysis in our assays, we used inhibitors of HOCl-modified protein induced platelet activation and specific inhibitors against α-defensins. HNP-induced platelet activation is inhibited by serpins^25^, which are thought to form complexes with HNPs causing inactivation of both binding partners^50^. As shown here, the serpins antithrombin III and α_1_-antitrypsin inhibited PVL-induced platelet activation in the presence of isolated neutrophils. Interestingly, it has been shown that rapid antithrombin III depletion is predictive for fatal outcome in patients with sepsis^51,52^. Therefore, especially in sepsis due to PVL-carrying *S*. *aureus*, antithrombin III might have another mode of action besides the well-characterised thrombin inhibition. Although, the phase III clinical antithrombin trial in patients with severe sepsis (KyberSept) failed^53^, those patients who were not given concomitant heparin showed significant mortality reduction^54^. Overall, this implies additional antithrombotic actions of antithrombin III, independent of the classical heparin-assisted inhibition of thrombin. Our present findings provide a rationale to rethink the clinical application of antithrombin III in a highly select patient group.

A further HNP inhibitor is SAK, a thrombolytic protein secreted by *S*. *aureus*, which forms complexes with HNPs^29,30^. As shown here, SAK also blocked PVL-induced platelet activation in the presence of isolated neutrophils. Therefore, *S*. *aureus* strains with high PVL and low SAK production might be especially detrimental with respect to thrombosis development. As defensin inhibitors block platelet activation by PVL, PVL-induced release of HNPs by neutrophils might make an important contribution to the observed activation of platelets.

Aside from HNPs, reactive oxygen species (ROS) such as HOCl generated by neutrophil myeloperoxidase, and proteins modified by HOCl also promote platelet activation^55,56^. The antioxidant GSH is the major scavenger for HOCl in mammalian cells^33,57^ and inhibits platelet activation by HOCl modified proteins (unpublished data, presented in abstract form)^34^. In this present study, GSH completely inhibited PVL-induced platelet activation in the presence of neutrophils. Resveratrol, an antioxidant naturally occurring in grapes, also has the ability to scavenge HOCl^58^. In addition, resveratrol is a known direct inhibitor of myeloperoxidase^35^ and of HOCl-modified proteins (unpublished; presented in abstract form)^34^. Therefore, just like GSH, resveratrol abolished platelet activation secondary to PVL-induced neutrophil lysis.

To examine if antioxidants may, in addition to their direct effects on platelets, also interfere with PVL-triggered neutrophil secretion, we determined the impact of resveratrol and the HOCl-scavenger taurine on neutrophil release of HNP-1–3, MPO, and nucleosomes, and the formation of acrolein-bound proteins. Taurine (2-aminoethanesulfonic acid) interacts directly with HOCl, resulting in the formation of the less toxic taurine chloramine (TauCl)^59^. In contrast to resveratrol, taurine does not inhibit platelet activation by HOCl-modified proteins (unpublished data, presented in abstract form)^34^. Thus, although taurine can interfere with newly-formed and secreted HOCl, it cannot modulate proteins that have already reacted with the radical. Accordingly, we consistently observed a stronger inhibitory effect of resveratrol in comparison with taurine. Overall, this study shows that the antioxidants resveratrol and taurine can suppress PVL-stimulated secretion of neutrophil-derived defensins, MPO, and nucleosomes, a surrogate marker for NETs^48^. This raises the interesting question if the antioxidants may also be beneficial in settings of PVL-induced pulmonary and skin necrosis.

We were given the opportunity to obtain blood from a CGD patient to examine the contribution of ROS formation to PVL-induced release reactions from neutrophils and secondary platelet activation. Neutrophils from the CGD patient exhibited significantly reduced H_2_O_2_ production, but were still able to generate α-defensins and MPO. As a consequence, platelet activation induced by PVL-treated neutrophils was only modestly reduced; a similar observation was made when neutrophils were stimulated with the positive control PMA. Since H_2_O_2_ production per se has some influence on platelet activation^60^, ADP-responsiveness was also less pronounced in the CGD patient in comparison with the healthy control donor.

Only limited interpretation of the data presented here is possible, given that we had access to a limited volume of blood from one patient only. It would be of great interest to compare PVL-induced neutrophil responses and secondary platelet activation in patients with CGD, and patients with MPO deficiency, given that HOCl appears to contribute critically to NET release. In the absence of functional NADPH oxidase activity, CGD neutrophils lack the capacity for NET formation in response to PMA, but NET formation is still inducible with HOCl^61^. Our results suggest that the PVL-induced neutrophil oxidative burst and myeloperoxidase release contributes to subsequent platelet activation in the same way that defensin secretion does. Most importantly, PVL failed to induce platelet activation in the presence of neutrophils when cells were isolated from blood after intravenous administration of reduced GSH. The results from the *ex vivo*/*in vitro* experiment support our hypothesis on the pathogenic mechanisms underlying thrombosis development in association with *S*. *aureus* osteomyelitis.

The effect of PVL on platelet activation was much less pronounced in whole blood than in platelet-rich-plasma or when we used gel-filtered platelets. This observation can be attributed to protease-inhibitors in the plasma. The whole blood used in our experiments was not neutrophil enriched, in contrast to the local accumulation of high amounts of neutrophils in osteomyelitis which are recruited to the site of infection. We assume that the protease-inhibiting effect of the plasma can be overcome by high amounts of reactive oxygen species as well as HNPs released by large numbers of damaged neutrophils.

In this work, we demonstrate that not only PVL can influence platelets secondary to neutrophils destruction, but that other bacterial toxins, such as PSMs, with a similar destructive effect on neutrophils, may also activate platelets. We found strong platelet activation by the supernatant of PSMα3 treated neutrophils. It must be noted that rather high and therefore supraphysiological concentrations of the toxin are required to induce neutrophil damage^16^ and, in a second step, to have an impact on platelets. Thus, a direct PSM effect on platelets is unlikely, although we did observe a modest direct effect of PSMα3 in this study. However, as shown by Hongo *et al*., PSMs are also able to augment the damaging effects of PVL on neutrophils^62^.

We found that neutrophil lysis by PVL could also be inhibited by antibodies in the human plasma. This is in accordance with our earlier study showing that antibodies in human serum protect neutrophils from lysis by PVL^24^. Additionally, here we show that platelet activation as a consequence of neutrophil damage was also inhibited by plasma with a high anti-PVL titre. Although DVT is generally relatively rare in children, cases associated with osteomyelitis have been repeatedly reported in the paediatric literature^20,63^. Until now there is no explanation why especially young patients are more likely to develop a DVT in association with *S*. *aureus* osteomyelitis than adult patients. Our results suggest that patients, who have developed a high antibody titre against PVL, are protected to some extent against complications associated with PVL-expressing *S*. *aureus*. Since in many countries the prevalence of PVL-expressing strains is still very low, the statistical probability for children to have already developed such a protective antibody titre is likely to also be low.

**Figure 8:**
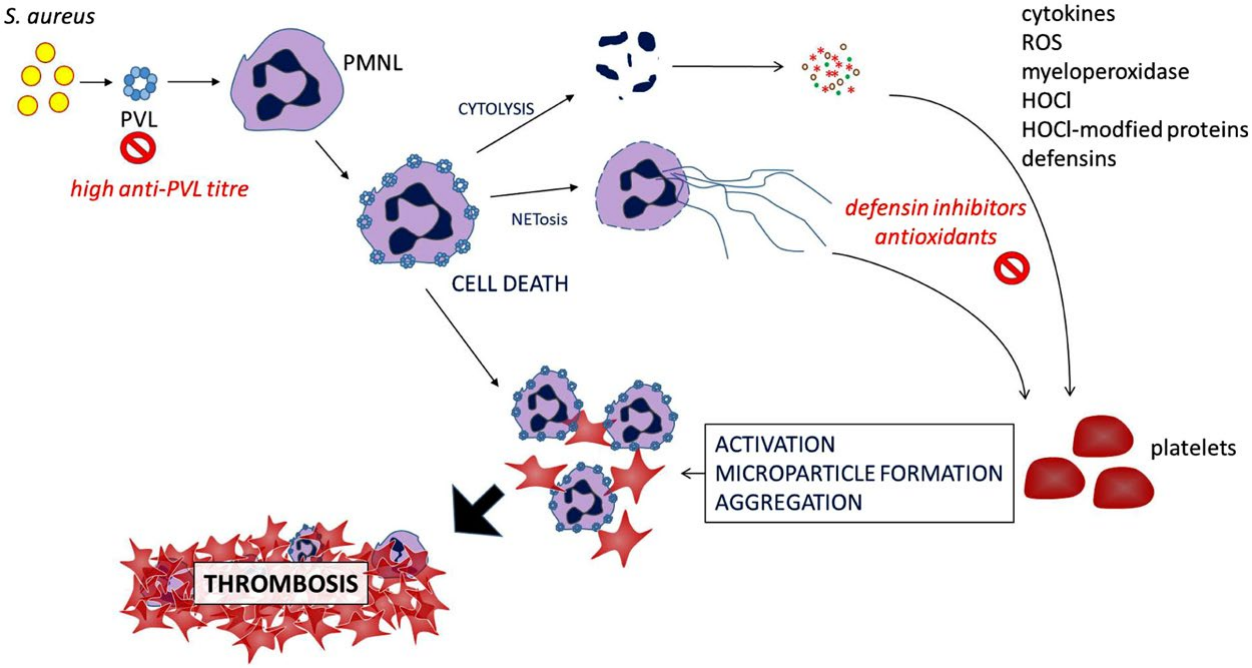
Simplified summary. PVL expressed by *S*. *aureus* induces neutrophil (PMNL) lysis and release of prothrombotic cytokines, defensins, myeloperoxidase, HOCl and HOCl-modified proteins, as well as NET formation. Subsequent platelet activation, microparticle release and aggregation ultimately drives thrombosis. High anti-PVL titre, defensin inhibition by serpins and staphylokinase and antioxidants glutathione and resveratrol are protective.

Taken together (see schematic summary in Fig. 8), we found that PVL, if not blocked by antibodies in the plasma, activated human platelets not directly, but only in the presence of high amounts of human neutrophils. This was mediated by binding of PVL to neutrophils, followed by lysis and release of HNPs and myeloperoxidase, with subsequent formation of HOCl-modification of proteins. Platelet activation by PVL-treated neutrophils will therefore depend on HNP release and the generation of HOCl-modified proteins which act as potent platelet agonists. This mechanism of platelet activation secondary to neutrophil lysis due to PVL might also be transferable to other neutrophil-targeting toxins. GSH, a scavenger of reactive oxygen species, inhibited platelet activation in the presence of PVL-damaged neutrophils. This inhibition was even observed *ex vivo* in blood collected after intravenous infusion with GSH. These observations should trigger thoughts about new approaches to treat and/or prevent thrombotic complications in the course of infections with PVL-producing *S*. *aureus* strains. These are urgently needed since the risk of infection with PVL-positive *S*. *aureus* strains is increasing, and many of these are MRSA strains with limited therapeutic options.

## Material and Methods

### Ethics statement

Human blood collection was conducted with approval of the local ethics committees (Ärztekammer Westfalen-Lippe und Medizinschen Fakultät der Westfälische Wilhelms-Universität Münster, WWU and Ärztekammer Nordrhein und Medizinische Fakultät der Universität Duisburg-Essen). All methods were performed in accordance with the relevant guidelines and regulations. Human blood samples were taken from healthy blood donors, who provided written informed consent for the collection of samples and subsequent platelet and neutrophil isolation and analysis.

### Materials

The conjugation of highly purified human fibrinogen with fluorescein isothiocyanate (FITC) via FITC-celite was performed as previously described^64^. Sytox-Green was from Molecular Probes and Hoechst33342 from Life Technologies. Anti-CD42a-PE, mouse IgG clone Beb1, anti-CD62P-FITC, PAC-1-FITC, and CD16b were from BD Pharmingen. Anti Mac-1 activation-dependent epitope antibody (CBRM1/5) was from eBioscience. Anti-human defensin antibody was purchased from Hycult Biotech and anti-FDP-lysine antibody was from Abcam. Anti-mouse IgG-FITC (Sigma-Aldrich) was used as secondary antibody. Resveratrol (≥98% trans-3,4′,5-Trihydroxystilben) was purchased from Carl Roth, human antithrombin III from Enzyme Research Laboratories, human serum, taurine, phorbol 12-myristate 13-acetate (PMA), bovine thrombin, ADP, Thrombin Receptor Activator Peptide 6 (TRAP-6) and α1-antitrypsin were from Sigma-Aldrich and reduced glutathione for i.v. injection (Tationil®) was from Roche (Italy). IgG depleted human serum was from Sunnylab. RPMI 1640 medium was from Biochrom. Melagatran (for injection) was from Astrazeneca (Wedel, Germany). Recombinant staphylokinase (SAK) was kindly provided by Dr. Bernhard Schlott (Leibnitz Institute for Age Research, Jena, Germany) and was prepared as previously described^65^.

### Expression and purification of recombinant PVL

PVL (LukS-PV and LukF-PV) was produced and purified as reported previously^16^. In brief, LukS-PV and LukF-PV were recombinantly expressed in *E*. *coli* TG1 using expression vectors for lukS-PV and lukF-PV. Cell lysates were used to purify the 6-His-tagged proteins by nickel-nitrilotriacetic acid affinity resin (Qiagen). In a last step buffer exchange was performed using Sephadex G-25 DNA grade columns to obtain LukS-PV and LukF-PV in phosphate-buffered saline (PBS).

### Preparation of human platelets

Blood was obtained with informed consent from healthy volunteers, who had not taken any medication affecting platelet function for at least 2 weeks before the study, and no non-steroidal anti-inflammatory drugs or antibiotics. Venous blood drawn from the antecubital vein was anticoagulated with trisodium citrate (0.0108 mol/L) and proceeded within 1 h after collection to prevent unspecific platelet activation and impairment of function. Platelet-rich plasma (PRP) was prepared by differential centrifugation at 200 × *g* for 10 min at room temperature. Platelets were resuspended in PBS. For some experiments, platelets were gel-filtered on Sephadex CL-2B as described earlier^66^. Gel-filtered platelets were resuspended in HEPES-Tyrode’s buffer supplemented with 2.5 mmol/L CaCl_2_. Platelets were counted using an automated cell counter (Baker 9020, Serono Diagnostics).

In one experiment, venous blood was drawn from the antecubital vein of 3 blood donors prior to and 30 min as well as 60 min after intravenous infusion of reduced glutathione. This was done by infusing one vial with 600 mg Tationil^®^iv. in 50 mL saline over 15 minutes into the arm vein. All blood donors receiving intravenous infusion of Tationil^®^ are senior authors of this study and gave written consent.

### Preparation of human neutrophils

Human neutrophils were isolated using discontinuous Percoll gradients as described previously^67^. Briefly, discontinuous Percoll gradients were created by layering 55% isotonic Percoll (GE Healthcare) onto 74% isotonic Percoll. The gradients were layered over with citrate-anticoagulated blood diluted 1:1 with sterile physiological saline. After centrifugation at 500 × g, the plasma-, platelet- and monocyte-containing upper phase was removed and discarded. Neutrophils, which were located at the interface between the 55% and the 74% Percoll layer, were collected, washed with Hank’s Buffered Salt Solution (HBSS) pH 7.4 and counted using an automated cell counter (Baker 9020, Serono Diagnostics). For experiments studying the neutrophil extracellular net formation, cells were resupended in RPMI buffer containing 0.5% human serum albumin after the last washing step. The purity of the neutrophil preparation was about 95%. Low activation status of neutrophils after isolation was verified by flow cytometry using an anti Mac-1 antibody (activation-dependant epitope).

### Binding of FITC-labelled PVL to platelets and neutrophils

LukS-PV and LukF-PV were labelled with FITC as described^68^. Briefly, proteins were incubated with a tenfold excess of FITC. The FITC-coupled PVL subunits were separated from free FITC by a G25 column. FITC-PVL concentration was determined using an Implen Nanophotometer (P330).

PMNLs (1 × 10^7^ per mL) ± gel-filtered platelets (2.5 × 10^7^ per mL) were incubated with FITC-labelled PVL (25 nmol/L) for 1 h at room temperature. After fixation with 1% paraformaldehyde and a washing step, cells were resuspended in PBS and labelled with anti-CD42a-PE for 30 min. After another washing step localization of PVL and antibody on the surface of platelets and neutrophils was analysed by fluorescence microscopy (Zeiss Observer.Z1).

### PMNL-platelet associate formation

Gel-filtered platelets (2.5 × 10^7^ cells/mL) together with isolated PMNLs (1 × 10^7^ cells/mL) were stimulated with PVL (0–50 nmol/L) for 1 h at room temperature. After fixation with 1% paraformaldehyde and staining of platelets with anti-CD42a-PE associate formation was determined by flow cytometric analysis, analysing the CD42a-PE-positive PMNLs.

### Preparation of bacteria

The staphylococcal strain USA300 and its isogenic deletion mutant USA300ΔPVL^16^ were grown in 10 mL of brain-heart infusion (BHI or BHI containing 150 µg/mL spectinomycin respectively) in a rotary shaker (160 rpm) at 37 °C for 15 h and pelleted for 10 min at 6,800 g. Supernatants were sterile-filtered through a 0.2 µmol/L Filtropur S-filter unit and used for the experiments.

### Flow cytometry

Agonist-induced binding of fibrinogen-FITC to platelets was performed as described previously^69^. Briefly, in the presence of 150 µg/mL fibrinogen-FITC and 2.5 mmol/L CaCl_2_, PRP or gel-filtered platelets diluted in buffer to a concentration of 2.5 × 10^7^ platelets per mL were incubated with PVL or bacterial supernatant for 60 min at room temperature with or without addition of isolated neutrophils, fixed with 1% paraformaldehyde and analysed with fluorescence-activated cell scanner (FACSCalibur, Becton Dickinson). Excitation occurred with an argon laser at 488 nm. FACSCalibur was used in a standard configuration with a 530-nm bandpass filter. Platelets were gated by FSC/SSC characteristics and data were obtained as median from fluorescence channel in a logarithmic mode. Platelets in whole blood were stained with anti CD42a-PE antibody (10 µL per 100 µL experimental sample) to be able to distinguish the platelets from other blood cells in flow cytometry. A total of 5,000 gated events were analysed for each data point. Inhibitors were pre-incubated with platelets for 10 min at room temperature previous to treatment with PVL. PAC-1 binding for the analysis of the activation of GPIIb/IIIa, and CD62P expression, as a marker for platelet activation with α−granule secretion, were quantified as reported earlier^25,70^. For the measurement of microparticles, gel-filtered platelets were stained with an anti CD42a-PE antibody (10 µL per 100 µL experimental sample) after incubation of platelets with PVL in the presence of neutrophils. Unstimulated and anti CD42a-PE stained platelets served as control. Anti-CD42a-PE positive events with reduced size in comparison to control cells, identified in a FFC/SSC-plot, were defined as microparticles and expressed as percentage of all anti-CD42a-PE-positive events measured.

To measure PVL-induced damage of neutrophils, freshly isolated human neutrophils (10,000 per µL) supplemented with 2.5 mmol/L CaCl_2_ were incubated with PVL for 1 h at room temperature. Neutrophils in whole blood were stained with CD16b and were gated by size scatter, allowing us to distinguish neutrophils from other blood cells during flow cytometry. Subsequently, samples were incubated with 12 µmol/L propidium iodide for 10 min and immediately analysed by flow cytometry. A total of 5,000 events were analysed for each data point. When inhibitors were tested, neutrophils were incubated with these substances for 10 min prior to stimulation with PVL.

### Preparation of neutrophil-conditioned supernatants for ELISA and platelet activation studies

Isolated neutrophils (10,000 per µL, in 500 µL) were incubated with PVL in the indicated concentrations, and in the presence of 2.5 mmol/L CaCl_2_, for 1 h at room temperature, ± study drugs and vehicle controls as indicated. Neutrophils in whole blood were incubated without addition of CaCl_2_. Conditioned supernatants were prepared by centrifugation at 1000 × g and immediately used for platelet activation studies or for quantification of myeloperoxidase (commercial kit from BioCheck Inc.), HNP1–3 (Kit from Hycult Biotech), NETs (Cell Death Detection Kit, Roche) or acrolein-bound proteins (FDP-Lysine/Acrolein-Lysine Adduct Competitive ELISA kit, Takara Bio Europe), according to the manufacturer’s instructions. To test the influence of reduced glutathione (GSH), neutrophils were treated with GSH for 10 min prior to stimulation with PVL. For platelet activation, PRP (5 × 10^7^ platelets/mL) was stimulated for 1 h at room temperature with conditioned PMNL supernatants in the indicated concentrations, in the presence of melagatran (3 μmol/L) to prevent secondary thrombin activation. Fibrinogen-FITC binding and platelet microparticles were then measured by flow cytometry analysis as described above.

### Quantification of the amount of antibodies against PVL in the plasma

Antibodies against PVL in the plasma were measured with a specific ELISA method with solid-phase LukF-PV and LukS-PV using a protocol adapted from Croze *et al*^23^. The wells of microtitre plates were coated with 125 µg/mL recombinant LukS-PV and LukF-PV in PBS overnight at room temperature. After blocking with 10% (w/v) skim milk in phosphate-buffered saline-Tween (0.05%) for 30 min at 37 °C, unbound LukS and LukF was washed out four times with the blocking solution. Serial dilutions of anti-PVL-antibody^16,24^ (1:100–1:100,000) for calibration, and plasmas (diluted 1:5,000 and 1:7,500) were added to wells for 1 h at 37 °C. After four washes horseradish peroxidase-conjugated goat anti-human IgG (1:2,500) (Promega) was added and the microplates were incubated for 1 h at 37 °C. After a washing step the substrate o-Phenylenediamine dihydrochloride (Sigma) was added and the plates were incubated in the dark for 30 minutes at room temperature. After incubation period the plates were read at 450 nm on a microplate reader. The results were expressed in arbitrary units.

### Depletion of anti-PVL-antibodies from human serum

Two columns containing nickel-nitrilotriacetic acid affinity resin were loaded with recombinant LukS-PV and LukF-PV. After washing the columns 4 times with PBS human serum was run 3 times through one column and afterwards 2 times through the other column. Anti PVL-antibody titres in the human serum before and after the run through the column were analysed by ELISA.

### Microscopic analysis

Human neutrophils (5 × 10^6^/mL) in RPMI-buffer were seeded in a 24-well plate on glass cover slips, activated for 45 min with 25 nmol/L PVL and fixed in 4% paraformaldehyde. The glass cover slips with the attached cells were washed with PBS, incubated with 4′,6-Diamidino-2-Phenylindole (DAPI) and an anti-defensin antibody, or an anti-FDP-lysine antibody to detect acrolein, which is formed through oxidative reactions, such as myeloperoxidase catalysed amino acid oxidation^22^. After a washing step and incubation with anti-mouse-IgG FITC the cells were analysed in a Nikon fluorescence microscope. Isotype controls were performed using mouse IgG Clone number 5F6.

For microscopic detection of cell death and NET formation, neutrophils (400 µL, 5 × 10^6^/mL) in RPMI supplemented with 0.5% human serum albumin were seeded into cell-culture dishes with cover glass bottom (MoBiTec) for 30 min. Neutrophils were then stimulated with PVL (1–50 nmol/L) at room temperature for 45 min, or with 100 nmol/L PMA for 2 h at 37 °C, 5%CO_2_, followed by addition of Sytox green (1 µmol/L) and Hoechst33342 (5 µg/mL) for a further 15 min in the dark. Cells were than directly imaged using a Zeiss Oberver.Z1.

### Statistical analysis

The results are expressed as mean ± standard deviation (SD). Statistical analyses were performed with Prism (GraphPad Software), using two-tailed unpaired t-test or one-way analysis of variance (ANOVA), followed by Bonferroni or Dunnett’s multiple comparison procedure vs. control as appropriate. Pearson’s correlation coefficient was used to determine the relationship between the percentage of undamaged neutrophils and anti-PVL-antibody titre. P < 0.05 was accepted as significant.

### Data availability

The authors declare that all the relevant data supporting the findings of the study are available in the article and its Supplementary Information files, or from the corresponding author upon request.

## Electronic supplementary material

Supplementary Information
