# Supplementary material for: Panton-Valentine Leukocidin associated with S. aureus osteomyelitis activates platelets via neutrophil secretion products
Source: Sci Rep. 2018 Feb 1;8:2185. doi: 10.1038/s41598-018-20582-z (PMC5794969; doi:10.1038/s41598-018-20582-z)
Supplement: Supplementary file 1 — Supplementary Information [file 41598_2018_20582_MOESM1_ESM.doc]

**Supplementary figures and figure legends**

**Panton-Valentine Leukocidin associated with S. aureus osteomyelitis activates platelets via neutrophil secretion products**

Silke Niemann^1+^, Anne Bertling^2,3+^, Martin F. Brodde^2^, Anke C. Fender^2^, Hélène Van de Vyver^1^, Muzaffar Hussain^1^, Dirk Holzinger^4^, Dirk Reinhardt^4^, Georg Peters^1,5^, Christine Heilmann^1, 3^, Bettina Löffler^6^ & Beate E. Kehrel^2,3*^

^1^Institute of Medical Microbiology, University of Muenster, Muenster, Germany;

^2^Department of Anaesthesiology, Intensive Care and Pain Medicine, Experimental and Clinical Haemostasis, University of Muenster, Muenster, Germany;

^3^Interdisciplinary Center for Clinical Research (IZKF) Muenster, Muenster, Germany;

^4^Department of Pediatric Hematology-Oncology, University of Duisburg-Essen, Essen, Germany;

^5^Cluster of Excellence EXC 1003, Cells in Motion, Muenster, Germany,

^6^Institute of Medical Microbiology, Jena University Hospital, Jena, Germany.

+ These two authors contributed equally to this work

***Corresponding author:** Prof. Dr. Beate E. Kehrel, Department of Anaesthesiology, Intensive Care and Pain Medicine, Experimental and Clinical Haemostasis, University of Muenster, Mendelstr. 11, 48149 Muenster, Germany; tel: +49(0)251-8356725; fax: +49(0)251-8352441; email: kehrel@uni-muenster.de

**a b**

**
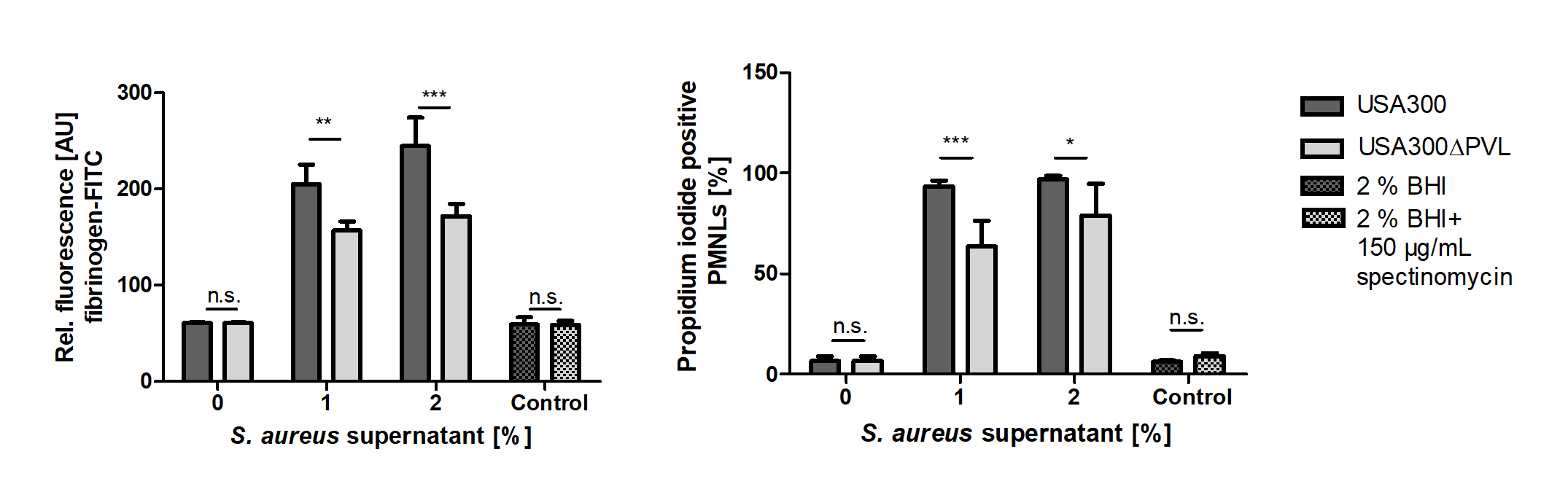
**

Supplementary Figure S1: Supernatant of PVL-producing *S. aureus* strain functionally resembled the recombinant PVL. Flow cytometric analysis of the effect of *S. aureus* USA300 or USA300ΔPVL supernatant in the presence of isolated PMNLs on fibrinogen-FITC-binding to gel-filtered platelets (a). Flow cytometric analysis of cell damage induced by the supernatant of *S. aureus* USA300 or *S. aureus* USA300ΔPVL by propidium iodide staining (b). BHI and BHI supplemented with 150 µg/mL spectinomycin served as vehicle control. Data are shown from 4 individual experiments, mean ± SD. Statistical significance was analyzed by two-way ANOVA followed by Bonfferoni posttests (* p < 0.05; ** p < 0.01; *** p < 0.001).


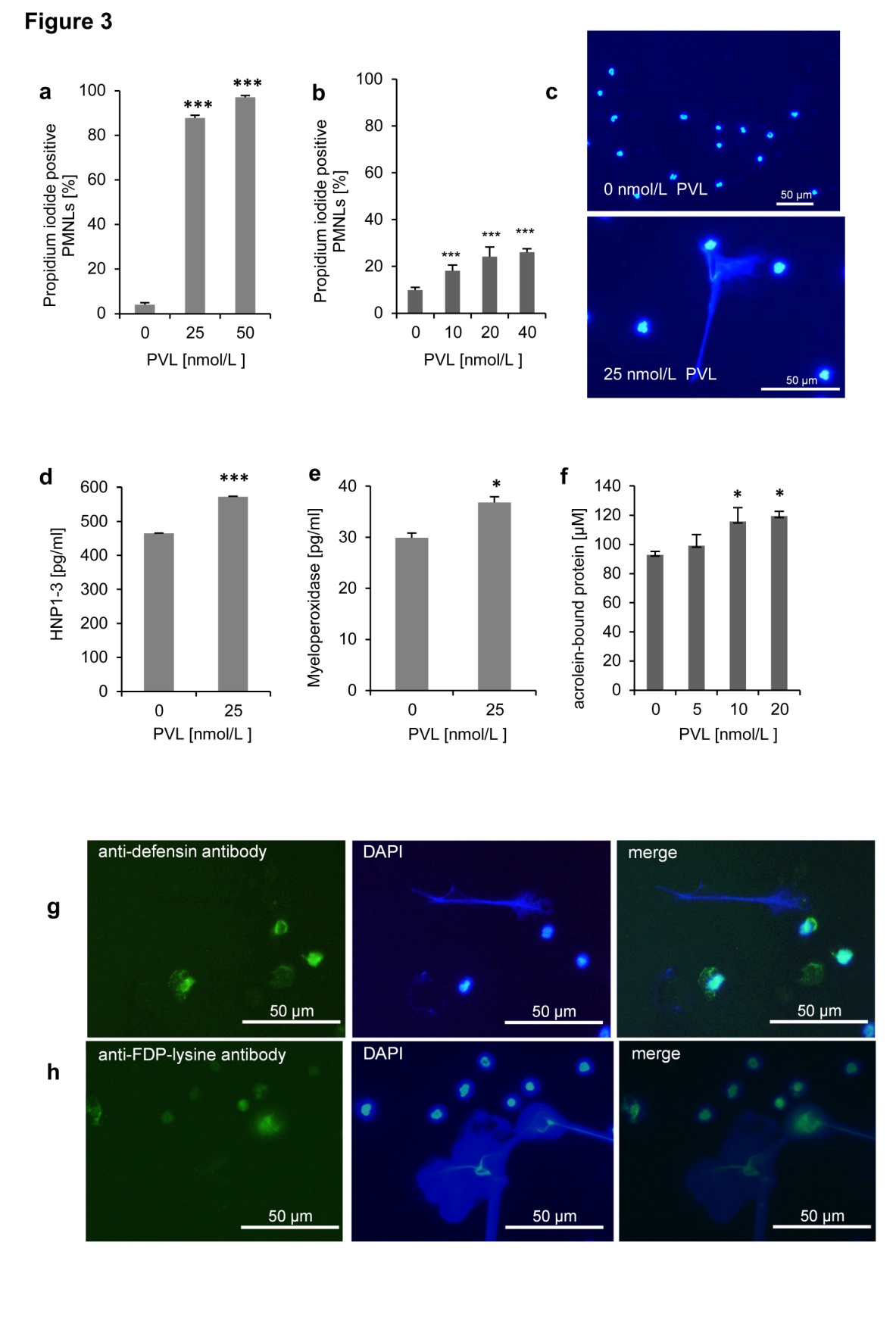
**25 nmol/L PVL,
45 min**


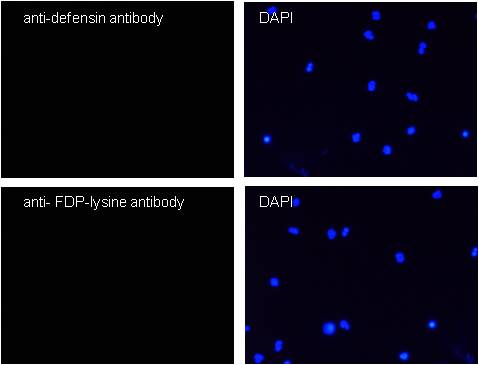


**0 nmol/L PVL**

**Supplementary Figure S2: Defensin-release and oxidative reactions by PVL-treated neutrophils.** Neutrophils were incubated with 25 nmol/L PVL for 45 min. HNP release was detected by an anti-defensin antibody (green) and oxidative reactions were detected by anti-FDP-lysine antibody directed against acrolein (green). DAPI was used as DNA stain (blue). Cells were fixed before staining. An overlay of the signals from DAPI staining and the respective antibodies is shown (blue and green, merge).

Supplementary figure S3: α1-antitrypsin, GSH and resveratrol does not inhibit platelet activation by ADP. Platelets in platelet rich plasma were activated by 10 µmol/L ADP in the presence or absence of α1-antitrypsin, GSH or resveratrol and in the presence of fibrinogen-FITC, followed by flow cytometric analysis. Data are shown from 3 individual experiments, mean ± SD. Statistical significance was analyzed by two-way ANOVA followed by Bonfferoni posttests.

Supplementary Figure S4: HNP-1 induced platelet fibrinogen binding is blocked by staphylokinase. Effect of 12 µmol/L and 60 µmol/L staphylokinase, respectively, on HNP-1 induced fibrinogen-FITC binding to gel-filtered platelets. After 5 minutes of preincubation with staphylokinase, platelets were stimulated with HNP-1 for 15 minutes in the presence of fibrinogen-FITC, followed by flow cytometric analysis. Data are mean ± SD from 3 different independent experiments; two-tailed unpaired Student’s t-test. *** p<0 .001 compared to the untreated control. HNP-1 human neutrophil peptide-1


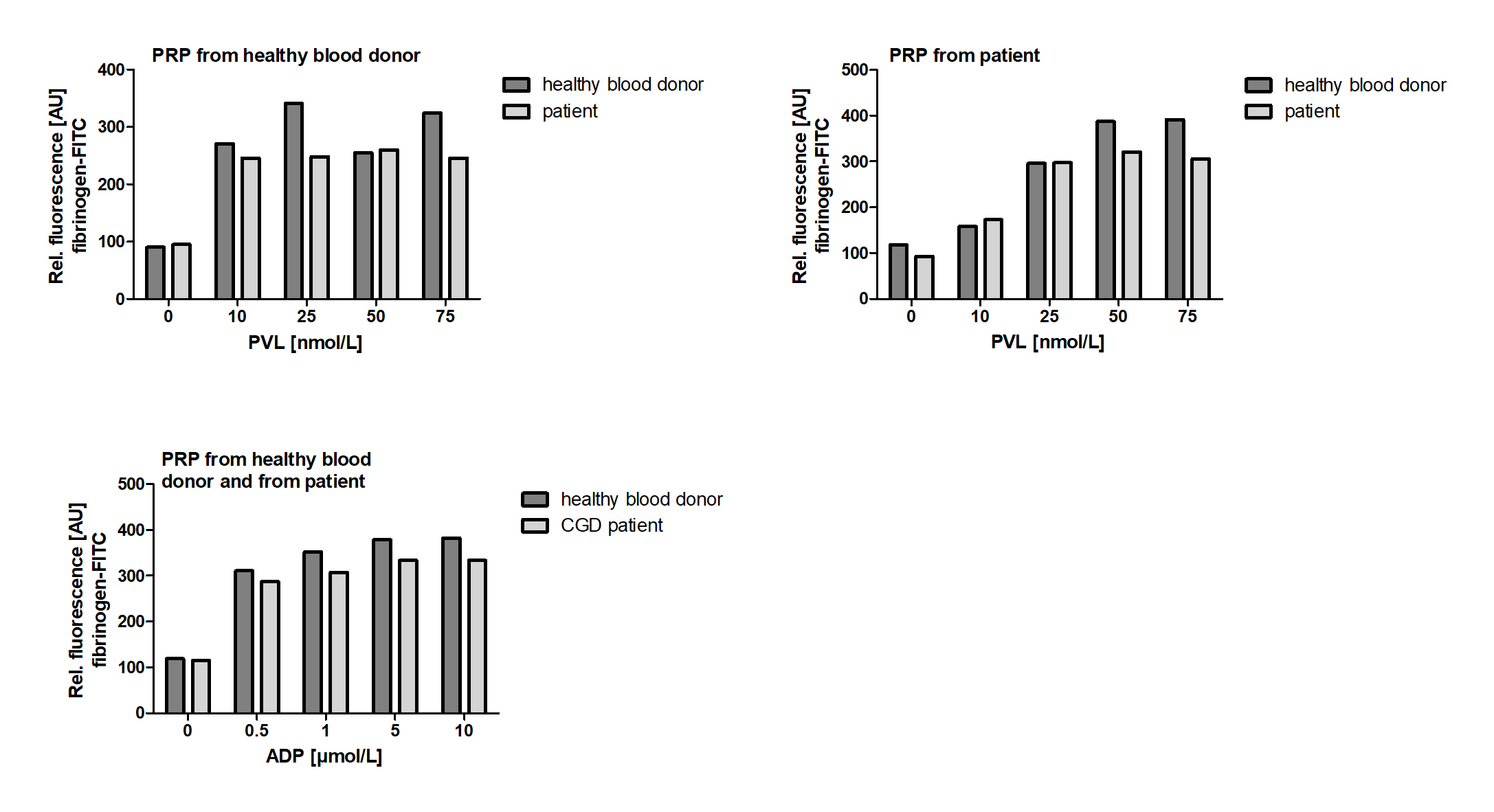
**a b**

**c**

**Supplementary Figure S5: Activation of platelets from CGD patient by ADP and by PVL in presence of PMNLs.** Flow cytometric analysis of the effect of PVL on fibrinogen-FITC-binding to platelets in platelet rich plasma (PRP) from a healthy blood donor (**a**) or from a CGD patient (**b**) in presence of isolated PMNLs from the patient or from a healthy blood donor. Platelets from CGD patient and from healthy blood donor were activated with ADP (**c**).


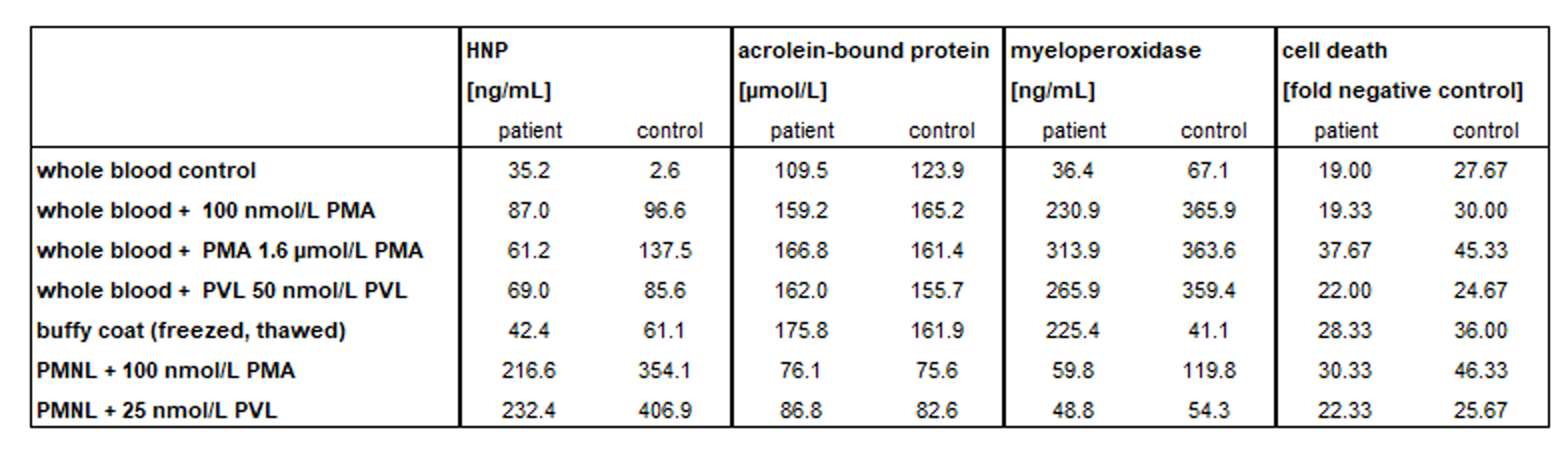


**Supplementary table 1: ELISA analyses of plasma samples obtained from whole blood after stimulation with either PVL or the positive control PMA .** Plasma samples were obtained from whole blood or from supernatant of neutrophils after stimulation with 50 nmol/L PVL, 100 nmol/L PMA or 1.6 µmol/L PMA. Samples were analysed by ELISA for: alpha-defensins HNP1-3 release, acrolein-bound protein formation, myeloperoxidase (MPO) release, or nucleosomes (cell death) as a surrogate marker for NET formation.


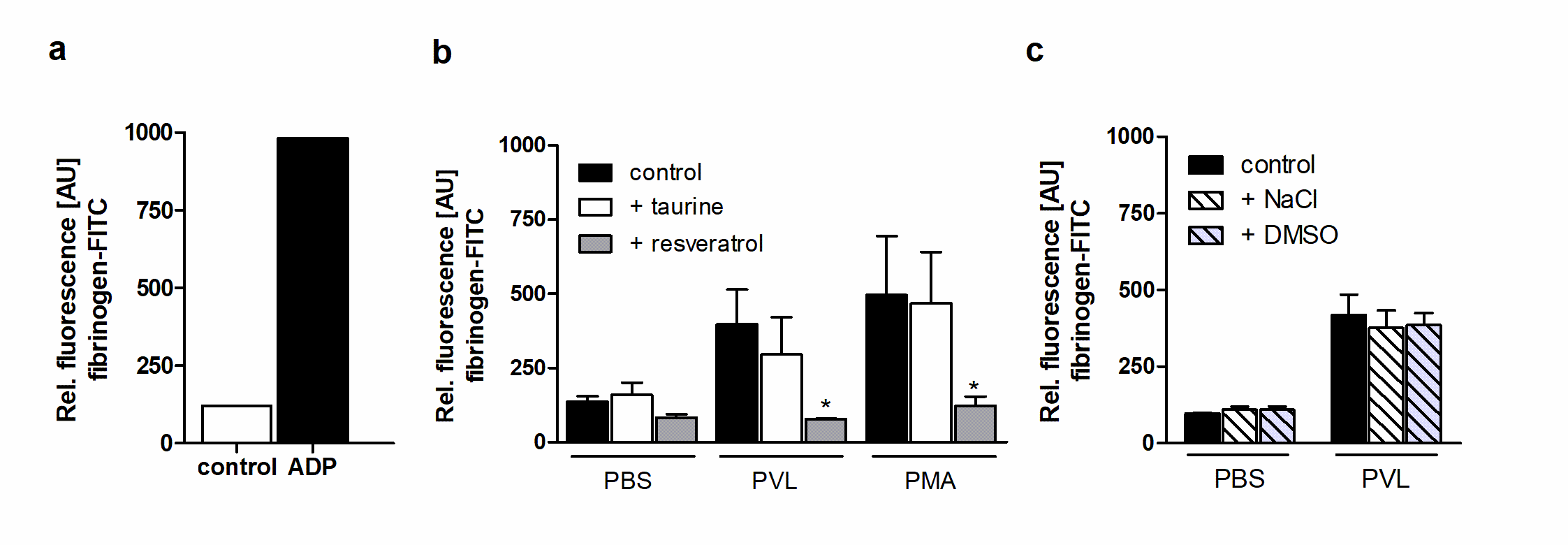


**Supplementary Figure S6: Secondary platelet activation by PVL-damaged neutrophils is reduced by antioxidants.** Platelet fibrinogen-FITC binding in PRP induced by ADP (10 µmol/L, 1h) (**a**) or 20% conditioned supernatants from human neutrophils (1h), which had been stimulated ± PVL (25 nmol/L) or PMA (100 nmol/L), in the absence and presence of either taurine (10 mmol/L) or resveratrol (50 µmol/L), all for 1h (**b**). Effects of vehicle controls for taurine and resveratrol (NaCl (0.9%) and DMSO (0.05% in PBS) respectively) are shown in (**c**). Data show mean ± SD from 3 independent experiments. Statistical significance was analyzed by one-way ANOVA followed by Dunnett’s multiple comparisons test (* p < 0.05, vs PVL or PMA without antioxidant).


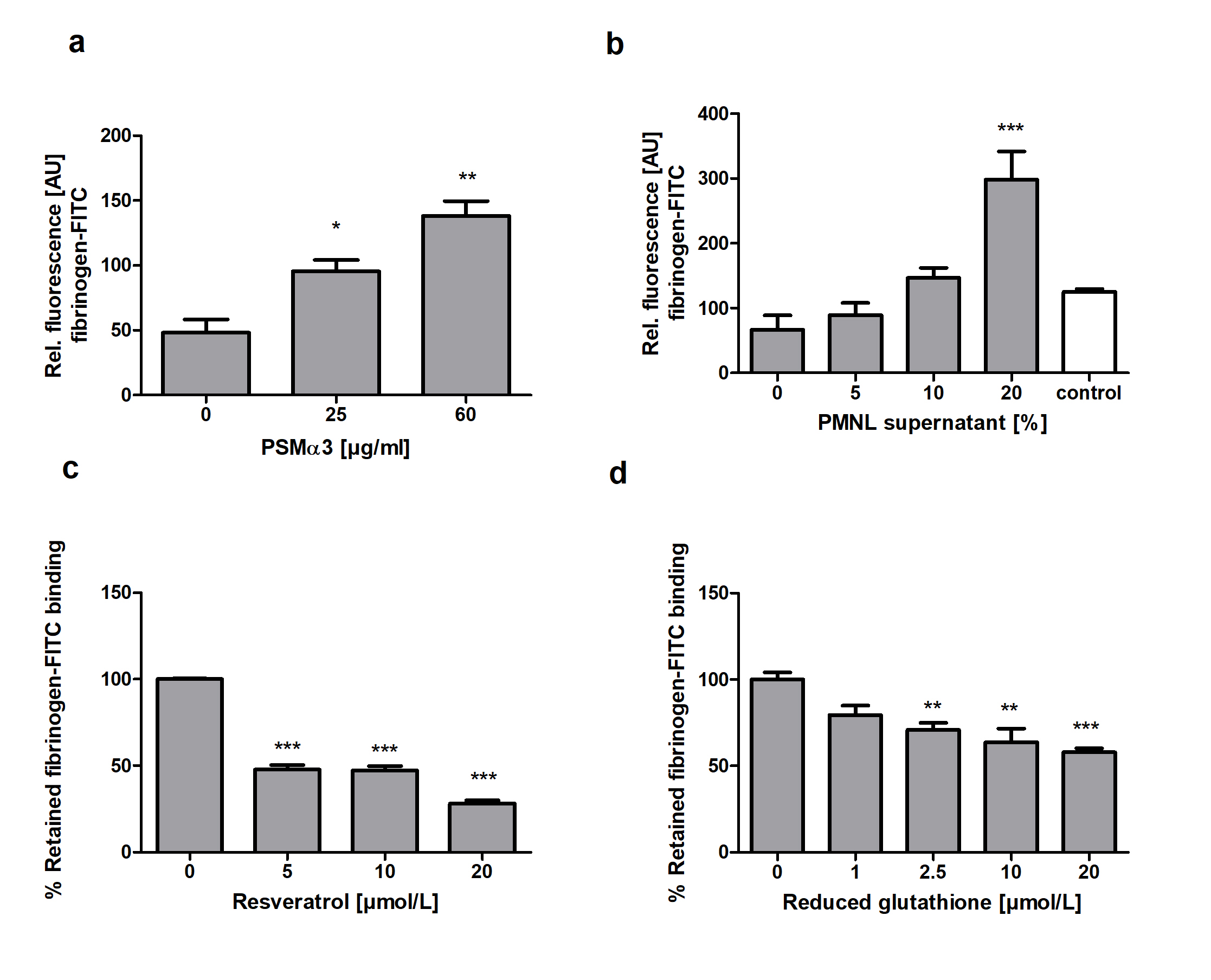


Supplementary Figure S7: Activation of platelets by PSMα3 and supernatant of PMNLs destroyed by ultrasound. Platelets were treated with PSMα3 for 1h. Fibrinogen-FITC binding was analysed by flow cytometric analysis (a). 10,000 isolated PMNLs/µl were destroyed by ultrasound (3 pulses: 2 seconds long, amplitude 60%, 5 seconds break) then centrifuged (5600 x g). Platelets were subsequently incubated with the neutrophil supernatant for 1h and fibrinogen-FITC binding was analyzed by flow cytometric analysis (b). Platelet activation by lysed PMNL supernatant can be inhibited by resveratrol (c) or GSH (d). Flow cytometric data represent means + SD of three independent experiments. Statistical significance was analyzed by one-way ANOVA followed by Dunnett’s multiple comparisons test (* p < 0.05; ** p < 0.01; *** p < 0.001).

Supplementary Figure S8: HNP-1-induced lyses of *S. aureus* USA300. HNP-1-induced influx of propidium iodide in *S. aureus* USA300 cells was measured over time by flow cytometry. Data are mean ± SD from 3 different experiments. HNP-1 human neutrophil peptide 1.
